# Glaucoma and microglia-induced neuroinflammation

**DOI:** 10.3389/fopht.2023.1132011

**Published:** 2023-02-27

**Authors:** Makoto Ishikawa, Yukitoshi Izumi, Kota Sato, Taimu Sato, Charles F. Zorumski, Hiroshi Kunikata, Toru Nakazawa

**Affiliations:** ^1^ Department of Ophthalmology, Tohoku University Graduate School of Medicine, Sendai, Japan; ^2^ Department of Ophthalmic Imaging and Information Analytics, Tohoku University Graduate School of Medicine, Sendai, Japan; ^3^ Taylor Family Institute for Innovative Psychiatric Research, Washington University School of Medicine, St. Louis, MO, United States; ^4^ Center for Brain Research in Mood Disorders, Washington University School of Medicine, St. Louis, MO, United States; ^5^ Department of Psychiatry, Washington University School of Medicine, St. Louis, MO, United States; ^6^ Department of Advanced Ophthalmic Medicine, Tohoku University Graduate School of Medicine, Sendai, Japan; ^7^ Department of Retinal Disease Control, Tohoku University Graduate School of Medicine, Sendai, Japan

**Keywords:** glaucoma, neuroinflammation, microglia, NOD-like receptor pyrin domain containing 3 inflammasome, retinal ganglion cell damage

## Abstract

Glaucoma is a multifactorial neurodegenerative disease characterized by a progressive optic neuropathy resulting in visual field defects. Elevated intraocular pressure (IOP) is the greatest risk factor for the development of glaucoma, and IOP reduction therapy is the only treatment currently available. However, there are many cases in which retinal degeneration progresses despite sufficient control of IOP. Therefore, it is important to elucidate the pathophysiology of glaucoma that is resistant to current IOP lowering therapies. Experiments using animal glaucoma models show the relationships between microglial neuroinflammatory responses and damage of retinal ganglion cells (RGCs). Inhibition of neuroinflammatory pathways associated with microglial activation appears to be neuroprotective, indicating that microglia may be an important therapeutic target for RGC protection. In this review, we will focus on microglia-induced neuroinflammation in the pathogenesis of glaucoma to offer new insights into the possibility of developing novel neuroprotective therapies targeting microglia.

## 1 Introduction

Glaucoma is an age-related multifactorial neurodegenerative disease of the optic nerve, and the leading cause of blindness in the world (1). Clinically, glaucoma is characterized by irreversible visual field loss due to optic nerve damage. The pathogenesis of glaucoma involves specific damage to retinal ganglion cells (RGCs) (2). Elevated intraocular pressure (IOP) is the most important risk factor for the development of glaucoma (3, 4), and IOP reduction therapy is the only evidence-based treatment for glaucoma (4). However, elevation of IOP is not necessary for the development of glaucomatous damage and loss of visual field. In clinical practice, there are cases in which visual field narrowing due to glaucoma progresses even when intraocular pressure is significantly lowered. Thus, it is important to clarify the pathophysiology of glaucoma in patients who show resistance to IOP reduction therapy.

In addition to IOP elevation, many other factors in glaucoma can adversely affect RGC survival and induce apoptosis, ultimately resulting in glaucomatous optic neuropathy. These factors include blood flow disturbance (5), oxidative stress (6), mitochondrial dysfunction (7), inactivation of autophagy (8), aging (9), and microglia-mediated neuroinflammation (10), but many aspects of pathogenesis remain unknown.

Neuroinflammation is originally a defensive process of the retina against damage. However, severe inflammation can induce retinal damage that may exert neurotoxic effects. Microglial activation is one of the first events in glaucomatous neurodegeneration. In experimental animal models of glaucoma, elevated IOP may activate retinal microglia, which release pro-inflammatory cytokines to damage RGC (10). It is thought that IOP lowering therapy is ineffective for RGC damage caused by microglial activation. It has been previously reported that administration of minocycline to the DBA/2J mouse (genetic model of glaucoma) protects RGCs and improves optic nerve integrity by suppressing microglial activation (11). However, the underlying mechanisms must be clarified in order to regulate microglia and protect RGCs efficiently.

In this paper, we review current knowledge concerning roles of microglia and interaction with astrocytes in glaucoma, and explore the possibility of developing novel neuroprotective therapies targeting microglia.

## 2 Functions of microglia in the retina

Among three types of glial cells (microglia, astrocytes, and Müller cells) in the retina, microglia are involved in chronic retinal inflammation. Microglia are macrophage-like glial cells that are resident in the retina, and distributed in an orderly mosaic pattern across three layers (outer retinal layer, inner retinal layer, and optic nerve fiber layer) in the retina. Microglia are thought to derive from monocytes that enter the retina from the blood stream during development, and dynamically move their cellular projections even under physiological conditions (12), making physical contact with neurons and synapses and performing synaptic pruning to remove unnecessary synapses (13). However, it has not been clarified whether microglia also shape developing inhibitory circuits by pruning. Recently, Favuzzi et al. (2021) show that microglia expressing the GABA_B1_ receptor participate in synaptic pruning of inhibitory circuits *via* a similar complement (C1q)-dependent mechanism as shown in synaptic pruning in excitatory circuits (14).

Microglia have a ramified morphology with thin, branched projections in the physiological state, while retract their projections and change to an ameboid form under stress (15). For convenience, active microglia are sometimes broadly classified into M1 microglia, which release inflammatory chemical mediators and act in a neuropathic manner, and M2 microglia, which release neurotrophic factors and act in a neuroprotective manner (15). However, it is known that there are various intermediate types of microglia in different pathological conditions, such as those that change from one to the other and those that combine the properties of both in response to changes in cytokine environment (16). Recent studies have reported that neuroinflammation may induce polarization of reactive microglia toward M1 (17). This leads us to expect that modulating microglial polarization towards the M2 phenotype may be a potential therapeutic strategy to reduce neuroinflammation. It has been reported that melatonin could reduce neuroinflammation and promote the conversion of M1 microglia phenotype to M2, as evident by the decrease of proinflammatory cytokines including TNF-αor IL-1β (18, 19). In fact, urinary melatonin excretion is significantly lowered in glaucoma patient (20, 21).

In experimental glaucoma, it has been observed that M1 microglia can migrate to remove the damaged or dead cells. The removal of unnecessary waste products from healthy cells is an important function that is also involved in the maintenance of nerve tissue function and the promotion of axonal regeneration (12). One major way that microglia clean up damaged organelles and proteins aggregates is through autophagy. Xu et al. (2021) (22) reveal that microglial autophagy critically controls microglial metabolic and immune status and also modulates neuroinflammation and neuronal tau pathology (the accumulation of the abnormally hyperphosphorylated tau in neurofibrillary degeneration and dementia). However, whether insufficient microglial autophagy induces glaucomatous RGC impairment has been remained to be clarified.

## 3 Microglia-mediated apoptosis and glaucomatous retinal ganglion cells

**Figure 1:**
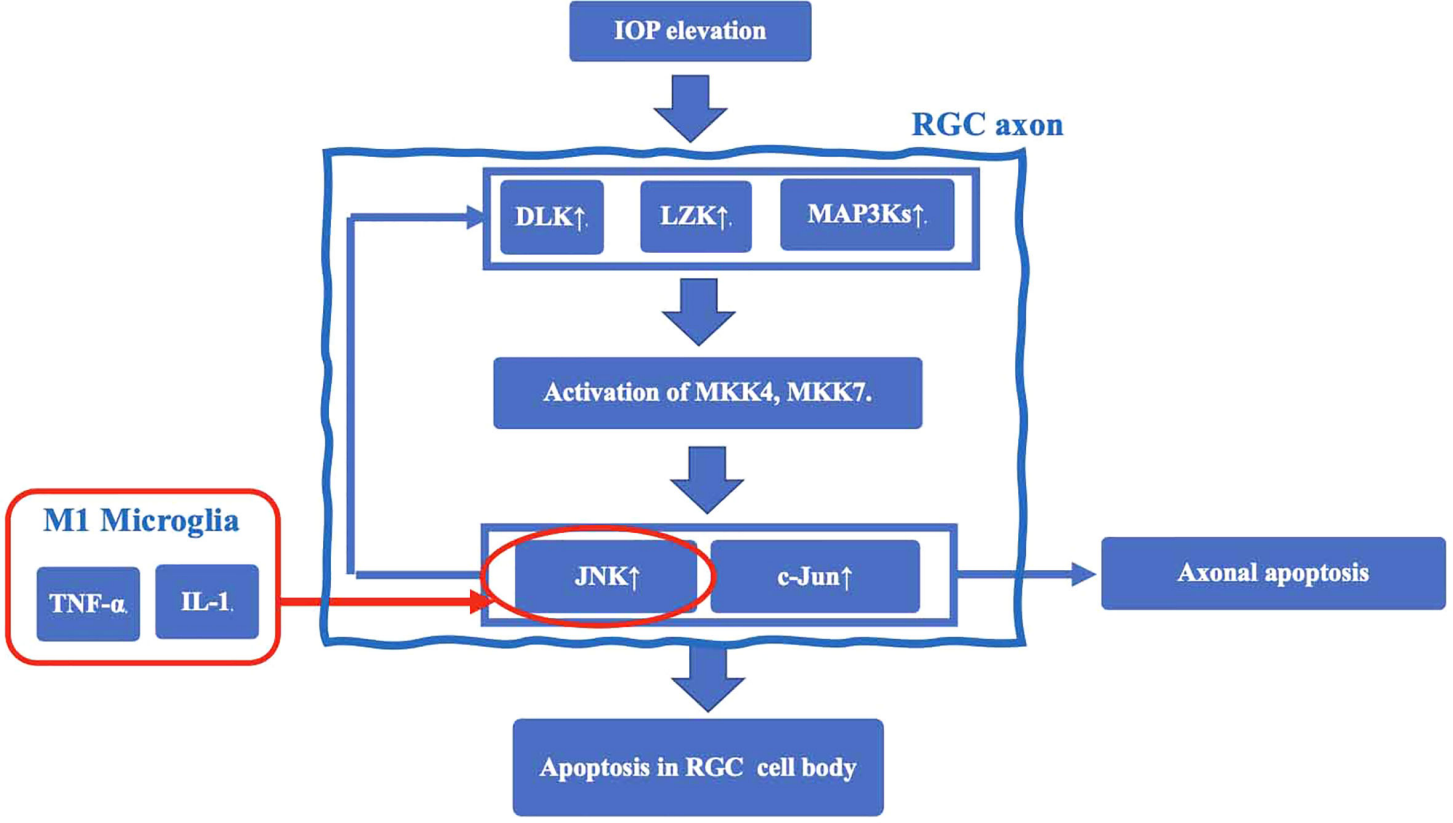
The mechanisms underlying RGC axonal injury by microglia. Elevated IOP induces expression of dual leucine zipper kinase (DLK), leucine zipper bearing kinase (LZK), and MAP3Ks, and activates MKK4 and MKK7 in RGC axons. This cascade results in upregulation of c-Jun N-terminal kinase (JNK) and the transcription factor c-Jun. JNK is an important mechanism of retrograde RGC injury. JNK is also activated by TNF-α and IL-1 released from M1 microglia to induce RGC apoptosis.

The earliest damage in glaucoma occurs in axons near the Lamina cribrosa (LC) of the optic nerve papilla (ONH), resulting in induction of apoptosis of RGC cell bodies (23). In a mouse glaucoma model, TNF-α activated microglial TNF receptor 2 (TNFR2) after elevated IOP. Simultaneously, oligodendrocytes decrease in the optic nerve and induces a further decrease in RGCs (24). The mechanisms underlying RGC axonal injury by microglia are thought to involve the following sequence (25): 1. Elevated IOP induces expression of dual leucine zipper kinase (DLK), leucine zipper bearing kinase (LZK), and MAP3Ks, and activates MKK4 and MKK7 in RGC axons. 2. As a result, c-Jun N-terminal kinase (JNK) and the transcription factor c-Jun, which not only stimulate axonal apoptotic signaling but also increase the expression level of DLK, are up-regulated, and induce apoptotic signaling in the RGC cell body. Thus, JNK is thought to be an important mechanism of so-called retrograde RGC injury, which starts from axonal damage and induces apoptosis. JNK is activated by TNF-α and IL-1 released from M1 microglia to induce RGC apoptosis. Taken together, a chronic inflammatory response mediated by TNF-α and IL-1 (25) may play an important role in the retrograde RGC damage pathway (**Figure 1**).

Microglial activation also associates with RGC apoptosis *via* lipid metabolism. Genome-wide association studies (GWAS) found that common variants near the ATP-binding cassette (ABC) transporter A1 (ABCA1) gene are associated with glaucoma (26–28). ABCA1 plays as a cholesterol efflux pump in the cellular lipid removal pathway, and an association between ABCA1 deficiency and retinal inflammation has been reported (29). Using a mouse model of ischemia-reperfusion (IR) induced by acute intraocular pressure (IOP) elevation, it has been revealed that IOP induced an increase in TANK-binding kinase 1 (TBK1) expression, which promotes ABCA1 ubiquitination and degradation, thus decreasing ANXA1 membrane transport and microglia activation, resulting in RGC apoptosis (29). These findings provide with ABCA1 and TBK1 novel targets for glaucoma therapies.

## 4 Production of proinflammatory cytokines by NLRP3 inflammasome in active microglia

Experimental animal models of Alzheimer’s disease revealed that the production of NOD-like receptor pyrin domain containing 3 (NLRP3) *via* Toll-like receptor 4 (TLR4) and the activation of the NLRP3 inflammasome *via* P2X7 purine receptor are essential for induction of neural inflammation (30). Consistently, it has been reported that RGC damage is substantially suppressed in NLRP3 knockout mice even when the optic nerve is damaged in experimental animal models of glaucoma (31). Although detailed mechanisms have not yet been clarified, TLR4 and P2X7 receptor-mediated response pathways may be closely related to RGC damage.

TLR4, which localizes to the plasma membrane of microglia, mediates innate immune responses. TLR4 is associated with both neuro-inflammation and clearance of protein aggregates in neurodegenerative disorders (32). With regard to TLR4 and glaucoma, it has been reported that a single nucleotide polymorphism in the TLR4 gene is associated with normal tension glaucoma and primary open-angle glaucoma (33). Intracellular substances such as proteins and fats released from damaged cells (damage associated molecular patterns, DAMPs) (34) and bacterial endotoxin, lipopolysaccharides (LPS) found in the outer membrane of gram-negative bacteria (35) specifically bind TLR4, and activate nuclear NF-κB signaling and generate NLRP3, which is a precursor of inflammatory cytokines, in the cytoplasm. Prointerleukin-1β (Pro-IL-1β) adaptor proteins and NLRP3s assemble into a characteristic heptameric structure and form a giant protein complex (the NLRP3 inflammasome) (36), which activates caspase-1 and produces the proinflammatory cytokines IL-1β and IL-18. These ILs in turn may damage RGCs.

It has been reported that administration of LPS in an experimental animal model of glaucoma worsened RGC damage through microglial activation mediated through TLR4 signaling and complement upregulation (37). Furthermore, it has also been found that chronic subclinical inflammatory reactions caused by oral bacteria that contain LPS in their outer membrane aggravate glaucoma (37), suggesting a possible involvement of LPS in NLRP3 inflammasome-mediated inflammatory reactions in glaucoma.

Adenosine triphosphate (ATP) is the primary energy source in all living organisms. When the cell membrane is disrupted (38), intracellular ATP is released outside the cell, where it is able to bind P2X7 receptors localized on microglial cell membranes. ATP then promotes an increase in intracellular K+ efflux, generation of reactive oxygen species (ROS), and lysosomal damage *via* the pannexin 1 receptor (39), resulting in activation of the NLRP3 inflammasome. Sakamoto et al. (2015) reported that BzATP, a P2X7 receptor agonist, had deleterious effect on the rat retina, and that A438079 and brilliant blue G, P2X7 receptor antagonists, reduced NMDA-induced retinal injury in the rat retina (40).

In a rat model of glaucoma, acute RGC injury caused by IOP elevation is mediated by endogenous extracellular ATP (41). Additionally, overexpression of purinergic P2X7 receptors contributes to death of RGCs in DBA/2J glaucomatous mice (42). Furthermore, JNJ47965567, a P2X7 receptor antagonist, preserves retinal ganglion cells, and improves pattern electroretinogram (ERG) signals in a murine glaucoma model (43). Patients with angle closure glaucoma have significantly higher levels of ATP in the anterior chamber than controls, and the level of ATP in the anterior chamber increases with IOP elevation (44).

However, we have to note that the localization of P2X7 receptors in the retina is not glia-specific (45). Ishii et al. (2003) (46) reported neuron (RGC and amacrine cell)-specific distribution of P2X7 receptors. Wheeler-Schilling et al. (2001) (47) reported expression of P2X7 receptors in RGC. Pannicke et al. (2000) (48) reported expression of P2X7 receptors in Müller glia. Sakamoto et al. (40) reported that immunohistochemical analysis demonstrated that P2X7 receptors were not expressed in the Iba1-positive microglial cells but in the somatic region of the RGCs in the rat retina.

Taken together, a series of studies indicates that TLR4 and P2X7 receptor-mediated response pathways may be closely related to RGC injury. Although the detailed relationship between the two pathways has not yet been elucidated, it is likely that ATP released from retinal neurons by IOP elevation binds microglial P2X7 receptors and efficiently activates the NLRP3 inflammasome generated *via* TLR4.

**Figure 2:**
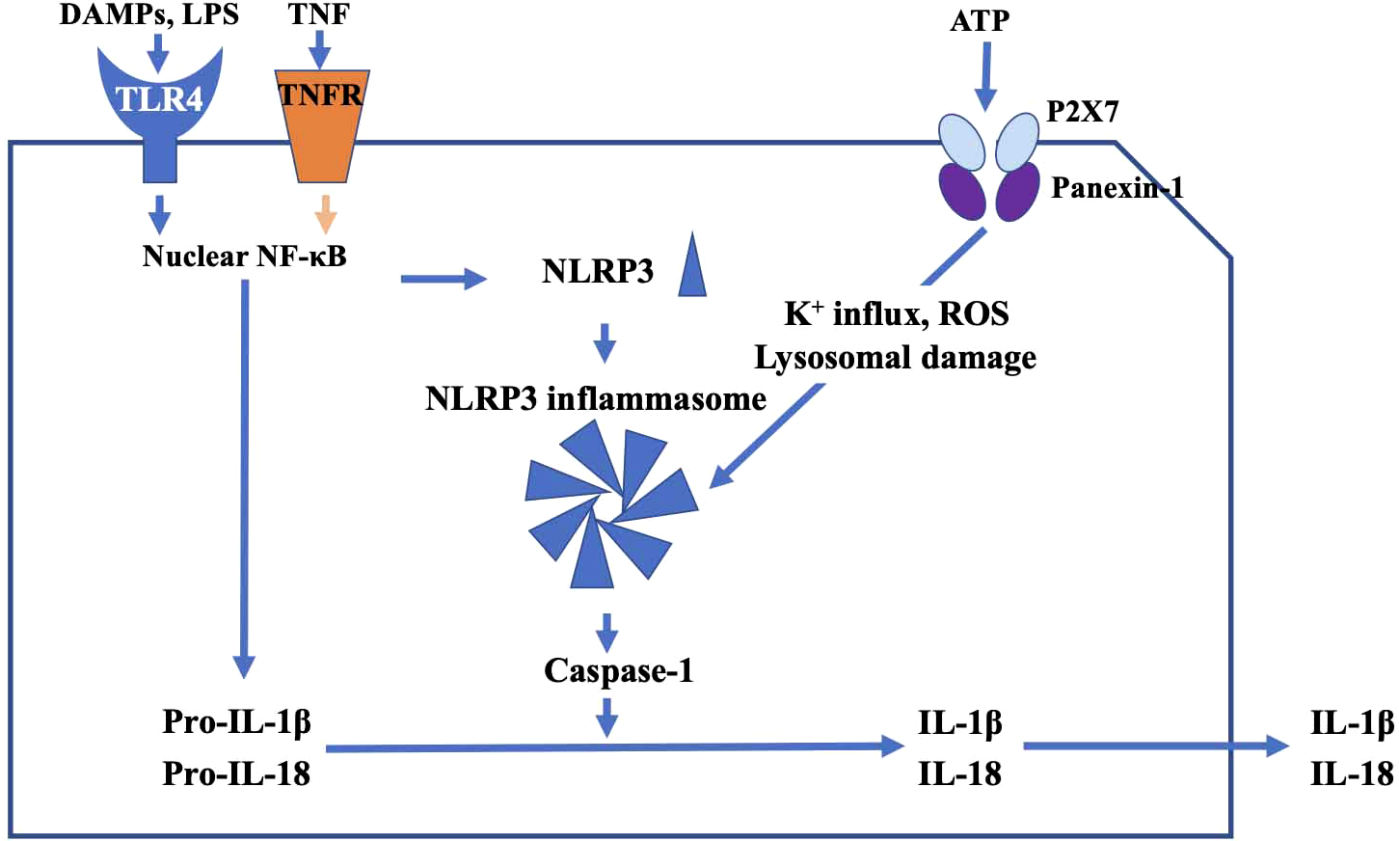
The main pathway of TLR4-mediated inflammatory response. DAMPs and TNF activate nuclear NF-κB signaling and generate NLRP3. Pro-IL-1β, adaptor proteins, and NLRP3 assemble into a characteristic heptameric structure and form the NLRP3 inflammasome. NLRP3 inflammasome activates caspase-1, and produces the proinflammatory cytokines IL-1β and IL-18, which in turn may damage RGCs. ATP promotes increase of intracellular K+ efflux, generation of reactive oxygen species (ROS), and lysosomal damage *via* the P2X7 receptor, resulting in activation of the NLRP3 inflammasome.

In addition to activation of the NLRP3 inflammasome by TLR4, tumor necrosis factor (TNF-α), which was originally reported as a factor that causes hemorrhagic necrosis of neoplastic tumors, is thought to induce inflammatory responses. TNF-α binds the TNF receptor (TNFR) of RGCs to induce apoptosis. TNF-α also upregulates expression of membrane Fas ligand (FasL) in microglia and stimulates Fas receptors in RGCs, leading to apoptosis initiated by caspase-8 activation (49). Recently, it has been reported that TNF-α could substitute for LPS as a priming signal, and activates the NLRP3 inflammasome *via* upregulation of NF-kB, and result in inflammasome-dependent IL-1β production in human primary macrophages (50, 51) (**Figure 2**).

In glaucoma patients, the TNF-α concentration in the anterior chamber is increased and the expression of TNF-α in retinal microglia and optic nerve astrocytes is elevated, while inhibition of TNF-α by drugs suppresses microglial activation, axonal degeneration and RGC loss (24). Thus, TNF-α appears to be an important potential therapeutic target for glaucoma.

## 5 Interaction between microglia and astroglia in glaucoma

In addition to microglia, astrocytes play an important role in maintaining retinal homeostasis and function. In response to retinal injuries and diseases, astrocytes can be activated into two types, a neurotoxic or pro-inflammatory phenotype (A1) and a neuroprotective or anti-inflammatory phenotype (A2). Astrocytes are transformed into the A1 phenotype by proinflammatory cytokines IL-1α, TNF-α, and C1q secreted by LPS-activated M1 microglia (41). A1 astrocytes do not contribute to neuronal survival, neuronal growth, synaptogenesis, or phagocytosis of synapses (52), but promote pro-inflammatory processes for the degradation of neurons and oligodendroglia (24, 53). Microglial secretion of IL-1α, TNF-α, and C1q was increased in a mouse glaucoma model (54), suggesting the possibility that microglia induce A1 astrocytes resulting in RGC damage in glaucoma. As A1 astrocytes and M1 microglia accompany neuroinflammation, it is difficult to distinguish between the contributions of A1astrocytes and M1 microglia in the neurodegenerative process.

Consistently, Guttenplan et al. (2020) (55) have demonstrated a significant decrease of RGC density after IOP elevation in the wild type mice, while such decrease in RGC number was prevented in an interleukin-1 (*Il1a)^-/-^/tumor necrosis factor alpha (Tnf)^-/-^/complement C1q (C1qa)^-/-^* mice which fail to produce proinflammatory cytokines following activation of microglia. These three cytokines induce neuroinflammatory reactive astrocyte which contribute to RGC death (55). These findings suggests that microglia-derived cytokines play a crucial role in microglia-to-astrocyte regulation in glaucoma (56).

Shinozaki et al. (2017) (57) have reported that that the downregulation of P2Y1 purinergic receptors, subclass of metabotropic P2Y receptors, by microglia-derived cytokines converts astrocytes to neuroprotective functions. These findings indicate the complexity of microglia-to-astrocyte regulation in neurodegenerative disorders. It is plausible that microglia can regulate reactive astrogliosis, and induce either neurotoxic or neuroprotective phenotype of astrocytes depending on the context of microglial activation (53).

Conversely, microglia are also known to be affected by astrocytes. In synaptic pruning by microglia, TGF-β secreted from astrocytes increases C1q expression at synaptic sites, and synapses tagged with complement proteins are eliminated by microglia (13). Although C1q expression is suppressed in the physiological state, its expression is upregulated in synaptic areas of the inner retinal reticular layer, leading to a subsequent decrease in RGCs and optic nerve fibers in a mouse model of glaucoma (DBA/2J mice) (58). Interestingly, in the retina of middle-aged (12 months old) C57BL/6J mice, it has been reported that exercise protects RGCs against dysfunction and cell loss after acute IOP elevation. This retinal protection was associated with preservation of inner retinal synapses and reduced synaptic complement deposition by the complement response (59). Exercise-induced protection may involve maintenance of brain-derived neurotrophic factor (BDNF) levels that overcome pressure-induced decreases in BDNF to dampen cell damage by Bcl-2 family members (such as Bax). The Bcl-2 family is known to promote apoptosis in the presence of BDNF deficiency (60). Significantly lower levels of BDNF have been detected in the sera and ocular fluids of glaucoma patients, indicating that neurotrophic deprivation is a likely mechanism of glaucomatous optic neuropathy.

## 6 Possible inhibitors of microglial toxicity for glaucoma treatment

Taken together, it is likely that microglial activation and secretion of pro-inflammatory cytokines are involved in glaucomatous retinal degeneration. Thus, agents that inhibit microglial toxicity have pharmaceutical potential against glaucoma. There are several candidates to modify microglial activation and pro-inflammatory processes. We discuss these agents in this section.

Minocycline is an inhibitor of microglial activation (61). Interestingly, glaucoma-like retinal degeneration induced by intravitreal injection of S100B in rats is partially prevented by prior intraperitoneal (i.p.) injection of minocycline (62). Ghrelin, a so called “hunger hormone”, also works as an inhibitor of microglial activation (63). In a rat glaucoma model, ghrelin reportedly prevented apoptosis, in spite of the fact that IOP elevation was not altered; ghrelin was also thought to act as an antioxidant in this study (64). Resveratrol, which is rich in red wine, is also an antioxidant but has a therapeutic potential as a modulator of microglial activation (65). In a rat glaucoma model, daily i.p. injection of resveratrol significantly preserved RGC densities over a 6 week period. Again, IOP elevation was not altered by this treatment, indicating that the neuroprotection is IOP independent (66). Curcumin, extracted from turmeric, is also an inhibitor of microglial activation (67). In a rat glaucoma model, topical application of curcumin twice-daily for three weeks significantly reduced RGC loss in an IOP independent manner. In this study, the problem of poor solubility of curcumin was resolved by formulation in a nanocarrier (68).

Candesartan is an angiotensin II type 1 receptor blocker used for the treatment of hypertension. Candesartan also inhibits TLR4 (69), and this effect would be expected to have anti-inflammatory actions. In a rat chronic glaucoma model, candesartan prevented RGC loss but did not lower IOP in the affected eyes during an observation period of 10 weeks (70). This report also indicates that orally active agents can be effective in treating glaucoma. Moreover, in excitatory amino acid carrier 1-deficient mice candesartan inhibits the increase in TLR4 activation in RGCs and protects RGCs (71), suggesting that this agent works not only against open angle glaucoma but also normal tension glaucoma (NTG) in which IOP lowering is less effective. These results further suggest that TAK-242, a specific inhibitor of TLR4, may have potential to protect RGCs in glaucoma. Although there are no studies indicating beneficiary actions against glaucoma, it has been reported that TAK-242 protects RGCs when it was administered intravitreally following optic nerve crush that damages axons akin to glaucoma (72).

Neurosteroids such as allopregnanolone (AlloP) are another intriguing set of agents that are neuroprotective and that may act *via* effects on neuroinflammation. AlloP is produced endogenously in the retina and endogenous AlloP helps to protect the retina from severe damage produced by high pressure in an *ex vivo* glaucoma model (73). However, endogenous AlloP is insufficient to protect RGCs and their axons completely in this *ex vivo* model, and full protection requires pharmacological doses. Exogenous AlloP is also highly protective following intravitreal injection in an *in vivo* glaucoma model, and acts *via* positive allosteric modulation of GABA-A receptors and stimulation of autophagy (8, 74). The protective effects of AlloP are independent of changes in IOP. Retinal protection by AlloP may also involve effects on microglia, and studies in both macrophages and brain indicate that it has anti-inflammatory effects *via* inhibition of TLR4-mediated signaling (75). AlloP also appears to inhibit TLR2 and TLR7, but not TLR3 (76). It is presently unknown whether anti-inflammatory effects of AlloP or effects on microglia contribute to neuroprotection in glaucoma models.

The agents described above are not specific inhibitors of microglial activation. For retinal protection there may be synergistic effects beyond inhibition of microglial activation. A common feature of these agents is that they do not alter IOP elevation, implying that it is more helpful if these agents are used together with regular drugs that control IOP.

## 7 Conclusion

Glaucoma is a multifactorial disease with complex interactions among multiple causes. Microglial activation is now thought to play an important role in RGC dysfunction and degeneration. Thus, regulation of microglial function could be a rational therapeutic approach to preserve RGCs from inflammatory cytokines in glaucomatous eyes, and inhibitors of microglial activation would represent a novel therapeutic direction for the treatment of glaucoma. Microglial inhibitors would not merely be an addition to currently available therapies to control IOP, but a promising neuroprotective approach to treat NTG which is poorly responsible to existing drugs.

## Author contributions

MI, YI, CZ, TS, TN wrote the original manuscript. KS and HK revised manuscript. All authors contributed to the article and approved the submitted version.

## References

[1] Tham YC Li X Wong TY Quigley HA Aung T Cheng CY. Global prevalence of glaucoma and projections of glaucoma burden through 2040: A systematic review and meta-analysis. Ophthalmology (2014) 121:2081–90. doi: 10.1016/j.ophtha.2014.11.030 24974815

[2] Quigley HA Dunkelberger GR Green WR. Chronic human glaucoma causing selectively greater loss of large optic nerve fibers. Ophthalmology (1988) 95:357–63. doi: 10.1016/s0161-6420(88)33176-3 3174003

[3] Quigley HA Broman AT. The number of people with glaucoma worldwide in 2010 and 2020. Bri. J Ophthalmol (2006) 90:262–7. doi: 10.1136/bjo.2005.081224 PMC1856963 16488940

[4] The AGIS Investigators. The advanced glaucoma intervention study (AGIS): 7. the relationship between control of intraocular pressure and visual field deterioration. Am J Ophthalmol (2000) 130:429–40. doi: 10.1016/s0002-9394(00)00538-9 11024415

[5] Kiyota N Shiga Y Omodaka K Pak K Nakazawa T. Time-course changes in optic nerve head blood flow and retinal nerve fiber layer thickness in eyes with open-angle glaucoma. Ophthalmology (2021) 128:663–71. doi: 10.1016/j.ophtha.2020.10.010 33065167

[6] Himori N Yamamoto K Maruyama K Ryu M Taguchi K Yamamoto M. Critical role of Nrf2 in oxidative stress-induced retinal ganglion cell death. J Neurochem (2013) 127:669–80. doi: 10.1111/jnc.12325 23721546

[7] Ju WK Liu Q Kim KY Crowston JG Lindsey JD Agarwal N. Elevated hydrostatic pressure triggers mitochondrial fission and decreases cellular ATP in differentiated RGC-5 cells. Invest. Ophthalmol Vis Sci (2007) 48:2145–51. doi: 10.1167/iovs.06-0573 17460273

[8] Ishikawa M Takaseki S Yoshitomi T Covey DF Zorumski CF Izumi Y. The neurosteroid allopregnanolone protects retinal neurons by effects on autophagy and GABRs/GABAA receptors in rat glaucoma models. Autophagy (2021) 17:743–60. doi: 10.1080/15548627.2020.1731270 PMC8032250 32070183

[9] Yu AL Birke K Moriniere J Welge-Lüssen U. TGF-{beta}2 induces senescence-associated changes in human trabecular meshwork cells. Invest. Ophthalmol Vis Sci (2010) 51:5718–23. doi: 10.1167/iovs.10-5679 20554622

[10] Tezel G. Molecular regulation of neuroinflammation in glaucoma: Current knowledge and the ongoing search for new treatment targets. Prog Retin. Eye Res (2021) 87:100998. doi: 10.1016/j.preteyeres.2021.1009 34348167 PMC8803988

[11] Bosco A Inman DM Steele MR Wu G Soto I Marsh-Armstrong N. Reduced retina microglial activation and improved optic nerve integrity with minocycline treatment in the DBA/2J mouse model of glaucoma. Invest. Ophthalmol Vis Sci (2008) 49:1437–46. doi: 10.1167/iovs.07-1337 18385061

[12] Kierdorf K Prinz M. Microglia in steady state. J Clin Invest. (2017) 127:3201–9. doi: 10.1172/JCI90602 PMC5669563 28714861

[13] Tremblay MÈ Stevens B Sierra A Wake H Bessis A Nimmerjahn A. The role of microglia in the healthy brain. J Neurosci (2011) 31(45):16064–9. doi: 10.1523/JNEUROSCI.4158-11.2011 PMC6633221 22072657

[14] Favuzzi E Huang S Saldi GA Binan L Ibrahim LA Fernández-Otero M. GABA-receptive microglia selectively sculpt developing inhibitory circuits. Cell (2021) 184(15):4048–4063.e32. doi: 10.1016/j.cell.2021.06.018 34233165 PMC9122259

[15] Varnum MM Ikezu T. The classification of microglial activation phenotypes on neurodegeneration and regeneration in alzheimer’s disease brain. Arch Immunol Ther Exp (Warsz) (2012) 60:251–66. doi: 10.1007/s00005-012-0181-2 PMC4429536 22710659

[16] Mikita J Dubourdieu-Cassagno N Deloire MS Vekris A Biran M Raffard G. Altered M1/M2 activation patterns of monocytes in severe relapsing experimental rat model of multiple sclerosis. amelioration of clinical status by M2 activated monocyte administration. Mult. Scler. (2011) 17:2–15. doi: 10.1177/1352458510379243 20813772

[17] Plastira I Bernhart E Goeritzer M Reicher H Kumble VB Kogelnik N. 1-oleyl-lysophosphatidic acid (LPA) promotes polarization of BV-2 and primary murine microglia towards an M1-like phenotype. J Neuroinflammat (2016) 13:205. doi: 10.1186/s12974-016-0701-9 PMC5002165 27565558

[18] Zhou Q Lin L Li H Wang H Jiang S Huang P. Melatonin reduces neuroinflammation and improves axonal hypomyelination by modulating M1/M2 microglia polarization *via* JAK2-STAT3-Telomerase pathway in postnatal rats exposed to lipopolysaccharide. Mol Neurobiol (2021) 58:6552–76. doi: 10.1007/s12035-021-02568-7 PMC8639545 34585328

[19] Kaur C Sivakumar V Robinson R Foulds WS Luu CD Ling EA. Neuroprotective effect of melatonin against hypoxia-induced retinal ganglion cell death in neonatal rats. J Pineal Res (2013) 54:190–206. doi: 10.1111/jpi.12016 23113620

[20] Kim JY Jeong AR Chin HS Kim NR. Melatonin levels in patients with primary open-angle glaucoma with high or low intraocular pressure. J Glaucoma. (2019) 28:154–60. doi: 10.1097/IJG.0000000000001130 30394980

[21] Yoshikawa T Obayashi K Miyata K Saeki K Ogata N. Decreased melatonin secretion in patients with glaucoma: Quantitative association with glaucoma severity in the LIGHT study. J Pineal Res (2020) 69:e12662. doi: 10.1111/jpi.12662 32333450

[22] Xu Y Propson NE Du S Xiong W Zheng H. Autophagy deficiency modulates microglial lipid homeostasis and aggravates tau pathology and spreading. Proc Natl Acad Sci U S A. (2021) 118:e2023418118. doi: 10.1073/pnas.2023418118 34187889 PMC8271658

[23] Howell GR Libby RT Jakobs TC Smith RS Phalan FC Barter JW. Axons of retinal ganglion cells are insulted in the optic nerve early in DBA/2J glaucoma. J Cell Biol (2007) 179:1523–37. doi: 10.1083/jcb.200706181 PMC2373494 18158332

[24] Nakazawa T Nakazawa C Matsubara A Noda K Hisatomi T She H. Tumor necrosis factor-α mediates oligodendrocyte death and delayed retinal ganglion cell loss in a mouse model of glaucoma. J Neurosci (2006) 26:12633–41. doi: 10.1523/JNEUROSCI.2801-06.2006 PMC6674838 17151265

[25] Syc-Mazurek SB Libby RT. Axon injury signaling and compartmentalized injury response in glaucoma. Retin. Eye Res (2019) 73:100769. doi: 10.1016/j.preteyeres.2019.07.002 PMC6898776 31301400

[26] Chen Y Lin Y Vithana EN Jia L Zuo X Wong TY. Common variants near ABCA1 and in PMM2 are associated with primary open-angle glaucoma. Nat Genet (2014) 46:1115–9. doi: 10.1038/ng.3078 25173107

[27] Gharahkhani P Burdon KP Fogarty R Sharma S Hewitt AW Martin S. Common variants near ABCA1, AFAP1 and GMDS confer risk of primary open-angle glaucoma. Nat Genet (2014) 46:1120–5. doi: 10.1038/ng.3079 PMC4177327 25173105

[28] Hysi PG Cheng CY Springelkamp H Macgregor S Bailey JNC Wojciechowski R. Genome-wide analysis of multi-ancestry cohorts identifies new loci influencing intraocular pressure and susceptibility to glaucoma. Nat Genet (2014) 46:1126–30. doi: 10.1038/ng.3087 PMC4177225 25173106

[29] Li L Xu L Chen W Li X Xia Q Zheng L. Reduced annexin A1 secretion by ABCA1 causes retinal inflammation and ganglion cell apoptosis in a murine glaucoma model. Front Cell Neurosci (2018) 12:347. doi: 10.3389/fncel.2018.00347 30364320 PMC6193130

[30] Thawkar BS Kaur G. Inhibitors of NF-κB and P2X7/NLRP3/Caspase 1 pathway in microglia: Novel therapeutic opportunities in neuroinflammation induced early-stage alzheimer’s disease. J Neuroimmunol. (2019) 326:62–74. doi: 10.1016/j.jneuroim.2018.11.010 30502599

[31] Puyang Z Feng L Chen H Liang P Troy JB Liu X. Retinal ganglion cell loss is delayed following optic nerve crush in NLRP3 knockout mice. Sci Rep (2016) 6:20998. doi: 10.1038/srep20998 26893104 PMC4759563

[32] Jack CS Arbour N Manusow J Montgrain V Blain M McCrea E. TLR signaling tailors innate immune responses in human microglia and astrocytes. J Immunol (2005) 175:4320–30. doi: 10.4049/jimmunol.175.7.4320 16177072

[33] Takano Y Shi D Shimizu A Funayama T Mashima Y Yasuda N. Association of toll-like receptor 4 gene polymorphisms in Japanese subjects with primary open-angle, normal-tension, and exfoliation glaucoma. Am J Ophthalmol (2012) 154:825–32. doi: 10.1016/j.ajo.2012.03.050 22831837

[34] McCarty MF Lerner A. The second phase of brain trauma can be controlled by nutraceuticals that suppress DAMP-mediated microglial activation. Expert Rev Neurother. (2021) 21:559–70. doi: 10.1080/14737175.2021.1907182 33749495

[35] Iannucci A Caneparo V Raviola S Debernardi I Colangelo D Miggiano R. Toll-like receptor 4-mediated inflammation triggered by extracellular IFI16 is enhanced by lipopolysaccharide binding. PloS Pathog (2020), 16 :e1008811. doi: 10.1371/journal.ppat.1008811 32903274 PMC7505474

[36] Halle A Hornung V Petzold GC Stewart CR Monk s Reinheckel T. The NALP3 inflammasome is involved in the innate immune response to amyloid-beta. Nat Immunol (2008) 9:857–65. doi: 10.1038/ni.1636 PMC3101478 18604209

[37] Astafurov K Elhawy E Ren L Don g Igboin C Hyman L. Oral microbiome link to neurodegeneration in glaucoma. PloS One (2014) 9:e104416. doi: 10.1371/journal.pone.0104416 25180891 PMC4152129

[38] Dong L Hu Y Zhou L Cheng X. P2X7 receptor antagonist protects retinal ganglion cells by inhibiting microglial activation in a rat chronic ocular hypertension model. Mol Med Rep (2018) 17:2289–96. doi: 10.3892/mmr.2017.8137 PMC5783460 29207073

[39] Moreira-Souza ACA Almeida-da-Silva CLC Rangel TP Rocha GDC Bellio M Zamboni DS. The P2X7 receptor mediates toxoplasma gondii control in macrophages through canonical NLRP3 inflammasome activation and reactive oxygen species production. Front Immunol (2017) 2017:1257. doi: 10.3389/fimmu.2017.01257 PMC5643413 29075257

[40] Sakamoto K Endo K Suzuki T Fujimura K Kurauchi Y Mori A. P2X7 receptor antagonists protect against n-methyl-D-aspartic acid-induced neuronal injury in the rat retina. Eur J Pharmacol (2015) 756:52–8. doi: 10.1016/j.ejphar.2015.03.008 25796199

[41] Resta V Novelli E Vozzi G Scarpa C Caleo M Ahluwalia A. Acute retinal ganglion cell injury caused by intraocular pressure spikes is mediated by endogenous extracellular ATP. Eur J Neurosci (2007) 25:2741–54. doi: 10.1111/j.1460-9568.2007.05528.x 17459106

[42] Pérez de Lara MJ Avilés-Trigueros M Guzmán-Aránguez A Valiente-Soriano FJ de la Villa P Vidal-Sanz M. Potential role of P2X7 receptor in neurodegenerative processes in a murine model of glaucoma. Brain Res Bull (2019) 150:61–74. doi: 10.1016/j.brainresbull.2019.05.006 31102752

[43] Romano GL Amato R Lazzara F Porciatti V Chou TH Drago F. P2X7 receptor antagonism preserves retinal ganglion cells in glaucomatous mice. Biochem Pharmacol (2020) 180:114199. doi: 10.1016/j.bcp.2020.114199 32798466

[44] Li A Zhang X Zheng D Ge J Laties AM Mitchell CH. Sustained elevation of extracellular ATP in aqueous humor from humans with primary chronic angle-closure glaucoma. Exp Eye Res (2011) 93:528–33. doi: 10.1016/j.exer.2011.06.020 PMC3374644 21745471

[45] Shinozaki Y Saito K Kashiwagi K Koizumi S. Ocular P2 receptors and glaucoma. Neuropharmacology (2023) 222:109302. doi: 10.1016/j.neuropharm.2022.109302 36341810

[46] Ishii K Kaneda M Li H Rockland KS Hashikawa T. Neuron-specific distribution of P2X7 purinergic receptors in the monkey retina. J Comp Neurol (2003) 459:267–77. doi: 10.1002/cne.10608 12655509

[47] Wheeler-Schilling TH Marquordt K Kohler K Guenther E Jabs R. Identification of purinergic receptors in retinal ganglion cells. Brain Res Mol Brain Res (2001) 92:177–80. doi: 10.1016/s0169-328x(01)00160-7 11483255

[48] Pannicke T Fischer W Biedermann B Schädlich H Grosche J Faude F. P2X7 receptors in müller glial cells from the human retina. J Neurosci (2000) 20:5965–72. doi: 10.1523/JNEUROSCI.20-16-05965.2000 PMC6772577 10934244

[49] Baudouin C Kolko M Parsadaniantz SM Messmer EM. Inflammation in glaucoma: From the back to the front of the eye, and beyond. Prog Retin. Eye Res (2021) 83:100916. doi: 10.1016/j.preteyeres.2020.100916 33075485

[50] Jämsen E Pajarinen J Kouri VP Rahikkala A Goodman SB Manninen M. Tumor necrosis factor primes and metal particles activate the NLRP3 inflammasome in human primary macrophages. Acta Biomat (2020) 108:347–57. doi: 10.1016/j.actbio.2020.03.017 PMC7729209 32194260

[51] Magni P Ruscica M Dozio E Rizzi E Beretta G Maffei Facino R. Parthenolide inhibits the LPS-induced secretion of IL-6 and TNF-alpha and NF-kappaB nuclear translocation in BV-2 microglia. Phytother. Res (2012) 26:1405–9. doi: 10.1002/ptr.3732 22359368

[52] Liddelow S Barres B. Reactive astrocytes: production, function, and therapeutic potential. Immunity (2017) 46:957–67. doi: 10.1016/j.immuni.2017.06.006 28636962

[53] Liddelow SA Guttenplan KA Clarke LE Bennett FC Bohlen CJ Schirmer L. Neurotoxic reactive astrocytes are induced by activated microglia. Nature (2017) 541:481–7. doi: 10.1038/nature21029 PMC5404890 28099414

[54] Sterling JK Adetunji JO Guttha S Bargoud AR Uyhazi KE Ross AG. GLP-1 receptor agonist NLY01 reduces retinal inflammation and neuron death secondary to ocular hypertension. Cell Rep (2020) 33:108271. doi: 10.1016/j.celrep.2020.108271 33147455 PMC7660987

[55] Guttenplan KA Stafford BK El-Danaf RN Adler DI Münch AE Weigel MK. Neurotoxic reactive astrocytes drive neuronal death after retinal injury. Cell Rep (2020) 31:107776. doi: 10.1016/j.celrep.2020.107776 32579912 PMC8091906

[56] Suzuki T Hide I Ido K Kohsaka S Inoue K Nakata Y. Production and release of neuroprotective tumor necrosis factor by P2X7 receptor-activated microglia. J Neurosci (2004) 24:1–7. doi: 10.1523/JNEUROSCI.3792-03.2004 14715932 PMC6729576

[57] Shinozaki Y Shibata K Yoshida K Shigetomi E Gachet C Ikenaka K. Transformation of astrocytes to a neuroprotective phenotype by microglia *via* P2Y1 receptor downregulation. Cell Rep (2017) 19:1151–64. doi: 10.1016/j.celrep.2017.04.047 28494865

[58] Stevens B Allen NJ Vazquez LE Howell GR Christopherson KS Nouri N. The classical complement cascade mediates CNS synapse elimination. Cell (2007) 131:1164–78. doi: 10.1016/j.cell.2007.10.036 18083105

[59] Chrysostomou V Galic S van Wijngaarden P Trounce IA Steinberg GR Crowston JG. Exercise reverses age-related vulnerability of the retina to injury by preventing complement-mediated synapse elimination *via* a BDNF-dependent pathway. Aging Cell (2016) 15:1082–91. doi: 10.1111/acel.12512 PMC5114604 27613664

[60] Almasieh M Wilson AM Morquette B Luis J Vargas C Polo AD. The molecular basis of retinal ganglion cell death in glaucoma. Prog Retin. Eye Res (2012) 31:152–81. doi: 10.1016/j.preteyeres.2011.11.002 22155051

[61] Grotegut P Perumal N Kuehn S Smit A Dick HB Grus FH. Minocycline reduces inflammatory response and cell death in a S100B retina degeneration model. J Neuroinflamm (2020) 17:375. doi: 10.1186/s12974-020-02012-y PMC7737388 33317557

[62] Grotegut P Kuehn S Meißner W Dick HB Joachim SC. Intravitreal S100B injection triggers a time-dependent microglia response in a pro-inflammatory manner in retina and optic nerve. Mol Neurobiol (2020) 57:1186–202. doi: 10.1007/s12035-019-01786-4 31705442

[63] Moon M Kim HG Hwang L Seo JH Kim S Hwang S. Neuroprotective effect of ghrelin in the 1-methyl-4-phenyl-1,2,3,6-tetrahydropyridine mouse model of parkinson’s disease by blocking microglial activation. Neurotox Res (2009) 15:332–47. doi: 10.1007/s12640-009-9037-x 19384567

[64] Can N Catak O Turgut B Demir T Ilhan N Kuloglu T. Neuroprotective and antioxidant effects of ghrelin in an experimental glaucoma model. Drug Des Devel Ther (2015) 9:2819–29. doi: 10.2147/DDDT.S83067 PMC4459614 26082612

[65] Maurya SK Bhattacharya N Mishra S Bhattacharya A Banerjee P Senapati S. Microglia specific drug targeting using natural products for the regulation of redox imbalance in neurodegeneration. Front Pharmacol (2021) 12:654489. doi: 10.3389/fphar.2021.654489 33927630 PMC8076853

[66] Pirhan D Yüksel N Emre E Cengiz A Kürşat Yıldız D. Riluzole- and resveratrol-induced delay of retinal ganglion cell death in an experimental model of glaucoma. Curr Eye Res (2016) 41:59–69. doi: 10.3109/02713683.2015.1004719 25658983

[67] Ding F Li F Li Y Hou X Ma Y Zhang N. HSP60 mediates the neuroprotective effects of curcumin by suppressing microglial activation. Exp Ther Med (2016) 12:823–8. doi: 10.3892/etm.2016.3413 PMC4950749 27446282

[68] Davis BM Pahlitzsch M Guo L Balendra S Shah P Ravindran N. Topical curcumin nanocarriers are neuroprotective in eye disease. Sci Rep (2018) 8:11066. doi: 10.1038/s41598-018-29393-8 30038334 PMC6056418

[69] Dasu MR Riosvelasco AC Jialal I. Candesartan inhibits toll-like receptor expression and activity both *in vitro* and *in vivo* . Atherosclerosis (2009) 202:76–83. doi: 10.1016/j.atherosclerosis.2008.04.010 18495130 PMC2676176

[70] Yang H Hirooka K Fukuda K Shiraga F. Neuroprotective effects of angiotensin II type 1 receptor blocker in a rat model of chronic glaucoma. Invest Ophthalmol Vis Sci (2009) 50:5800–4. doi: 10.1167/iovs.09-3678 19608537

[71] Semba K Namekata K Guo X Harada C Harada T Mitamura Y. Renin–angiotensin system regulates neurodegeneration in a mouse model of normal tension glaucoma. Cell Death Dis (2014) 5:e1333. doi: 10.1038/cddis.2014.296 25032856 PMC4123089

[72] Nakano Y Shimazawa M Ojino K Izawa H Takeuchi H Inoue Y. Toll-like receptor 4 inhibitor protects against retinal ganglion cell damage induced by optic nerve crush in mice. J Pharmacol Sci (2017) 133:176–83. doi: 10.1016/j.jphs.2017.02.012 28318829

[73] Ishikawa M Yoshitomi T Zorumski CF Izumi Y. Neurosteroids are endogenous neuroprotectants in an ex vivo glaucoma model. Invest Ophthalmol Vis Sci (2014) 55:8531–41. doi: 10.1167/iovs.14-15624 PMC4280088 25406290

[74] Ishikawa M Nakazawa T Kunikata H Sato K Yoshitomi T Krishnan K. The enantiomer of allopregnanolone prevents pressure-mediated retinal degeneration *Via* autophagy. Front Pharmacol (2022) 13:855779. doi: 10.3389/fphar.2022.855779 35370641 PMC8966700

[75] Balan I Beattie MC O’Buckley TK Aurelian L Morrow AL. Endogenous neurosteroid (3α, 5α)3-hydroxypregnan-20-one inhibits toll-like-4 receptor activation and pro-inflammatory signaling in macrophages and brain. Sci Rep (2019) 9:1220. doi: 10.1038/s41598-018-37409-6 30718548 PMC6362084

[76] Balan I Aurelian L Schleicher R Boero G O’Buckley T Morrow AL. Neurosteroid allopregnanolone (3α,5α-THP) inhibits inflammatory signals induced by activated MyD88-dependent toll-like receptors. Transl Psychiatry (2021) 11:145. doi: 10.1038/s41398-021-01266-1 33637705 PMC7909379

