# Corrigendum: Glaucoma and microglia-induced neuroinflammation

**DOI:** 10.3389/fopht.2023.1332312

**Published:** 2023-12-01

**Authors:** Makoto Ishikawa, Yukitoshi Izumi, Kota Sato, Taimu Sato, Charles F. Zorumski, Hiroshi Kunikata, Toru Nakazawa

**Affiliations:** ^1^ Department of Ophthalmology, Tohoku University Graduate School of Medicine, Sendai, Japan; ^2^ Department of Ophthalmic Imaging and Information Analytics, Tohoku University Graduate School of Medicine, Sendai, Japan; ^3^ Taylor Family Institute for Innovative Psychiatric Research, Washington University School of Medicine, St. Louis, MO, United States; ^4^ Center for Brain Research in Mood Disorders, Washington University School of Medicine, St. Louis, MO, United States; ^5^ Department of Psychiatry, Washington University School of Medicine, St. Louis, MO, United States; ^6^ Department of Advanced Ophthalmic Medicine, Tohoku University Graduate School of Medicine, Sendai, Japan; ^7^ Department of Retinal Disease Control, Tohoku University Graduate School of Medicine, Sendai, Japan

**Keywords:** glaucoma, neuroinflammation, microglia, NOD-like receptor pyrin domain containing 3 inflammasome, retinal ganglion cell damage

**Incorrect Reference**

In the published article, one of the references has been retracted. This article has been removed, and replaced with the following article: *Tremblay MÈ, Stevens B, Sierra A, Wake H, Bessis A, Nimmerjahn A. The role of microglia in the healthy brain. J Neurosci (2011) 31(45):16064-9. doi: 10.1523/JNEUROSCI.4158-11.2011*

The article text has also been updated to reflect this change. The first paragraph of Section 2, *Functions of Microglia in the Retina*, previously read as follows:

*“Microglia are thought to derive from monocytes that enter the retina from the blood stream during development, and dynamically move their cellular projections even under physiological conditions (12), making physical contact with neurons and synapses and performing synaptic pruning to remove unnecessary synapses. In the development of excitatory circuits, synaptic pruning, by which extrasynaptic connections are eliminated by microglia, is thought to occur when complement C1q is expressed at synapses by TGF-β secreted from astrocytes (13).”*

The corrected sentence appears below:

*“Microglia are thought to derive from monocytes that enter the retina from the blood stream during development, and dynamically move their cellular projections even under physiological conditions (12), making physical contact with neurons and synapses and performing synaptic pruning to remove unnecessary synapses (13).”*

The authors apologize for this error and state that this does not change the scientific conclusions of the article in any way. The original article has been updated.

